# Norepinephrine stimulates progesterone production in highly estrogenic bovine granulosa cells cultured under serum-free, chemically defined conditions

**DOI:** 10.1186/1477-7827-10-95

**Published:** 2012-11-22

**Authors:** Carla A Piccinato, Luis H Montrezor, Cristhianna AV Collares, Alessandra A Vireque, Alzira AM Rosa e Silva

**Affiliations:** 1 Hospital Israelita Albert Einstein, São Paulo, Brazil; 2 Department of Physiology, School of Medicine of Ribeirão Preto, Universidade de São Paulo, São Paulo, Brazil; 3 Barão de Mauá University, Ribeirão Preto, São Paulo, Brazil; 4 Department of Gynecology and Obstetrics, School of Medicine of Ribeirão Preto, Universidade de São Paulo, São Paulo, Brazil; 5 Department of Physiological Science, Biological Sciences Institute, Universidade de Brasília, Brasília, Brazil

**Keywords:** Norepinephrine, Granulosa cells, Progesterone, Estradiol, Catecholamines, PVA, Bovine, Steroidogenesis

## Abstract

**Background:**

Since noradrenergic innervation was described in the ovarian follicle, the actions of the intraovarian catecholaminergic system have been the focus of a variety of studies. We aimed to determine the gonadotropin-independent effects of the catecholamine norepinephrine (NE) in the steroid hormone profile of a serum-free granulosa cell (GC) culture system in the context of follicular development and dominance.

**Methods:**

Primary bovine GCs were cultivated in a serum-free, chemically defined culture system supplemented with 0.1% polyvinyl alcohol. The culture features were assessed by hormone measurements and ultrastructural characteristics of GCs.

**Results:**

GCs produced increasing amounts of estradiol and pregnenolone for 144h and maintained ultrastructural features of healthy steroidogenic cells. Progesterone production was also detected, although it significantly increased only after 96h of culture. There was a highly significant positive correlation between estradiol and pregnenolone production in high E2-producing cultures. The effects of NE were further evaluated in a dose–response study. The highest tested concentration of NE (10 (−7) M) resulted in a significant increase in progesterone production, but not in estradiol or pregnenolone production. The specificity of NE effects on progesterone productio n was further investigated by incubating GCs with propranolol (10 (−8) M), a non-selective beta-adrenergic antagonist.

**Conclusions:**

The present culture system represents a robust model to study the impact of intrafollicular factors, such as catecholamines, in ovarian steroidogenesis and follicular development. The results of noradrenergic effects in the steroidogenesis of GC have implications on physiological follicular fate and on certain pathological ovarian conditions such as cyst formation and anovulation.

## Background

The intraovarian regulatory system that modulates the response to gonadotropins and ovarian functions is complex. Neurotransmitters, such as catecholamines, have been considered as intraovarian regulators due to the identification of intrinsic catecholamine synthesis and the presence of extensive sympathetic innervation in the mammalian ovary [1-4]. In fact, the mammalian ovary is richly supplied by sympathetic fibers that innervate the interstitium and the perifollicular regions of developing follicles, including the external thecal layer, but do not reach the GC layer [3]. Although some minor species differences may exist regarding localization, distribution, and density, the sympathetic innervation of the ovary is overall similar in many animals [2,3,5], including ruminants [6]. Among the catecholamines, norepinephrine (NE) is the most abundant neurotransmitter released by sympathetic nerves in the mammalian ovary [7].

The proposed mechanism of action for NE in the ovary involves binding to ovarian adrenergic receptors [8,9], induction of cAMP, leading to follicular development [10]. In addition, few studies have evaluated the effects of NE on the synthesis of the key ovarian hormones progesterone (P4) and estradiol (E2). Norepinephrine has been previously shown to induce P4 production in the bovine corpus luteum [11,12]. However, the effects of NE in E2 production by ovarian follicular granulosa cells (GCs) have not been demonstrated in mammalian species, despite the known elevation of catecholamine levels in the estrogenic pre-ovulatory phase of the rodent estrous cycle [13] and the presence of high concentrations of NE in the follicular fluid and follicular wall of both, small antral and pre-ovulatory follicles of cows [14]. Although NE is known to stimulate ovarian steroidogenesis via a gonadotropin-mediated mechanism [9,13], there is little evidence of a direct mechanism of action of NE on specific steroid hormone synthesis. Moreover, the effects of NE in the highly steroidogenic growing follicle have not yet been studied. The lack of an appropriate model for testing both gonadotropin-dependent and -independent NE effects possibly explains our limited understanding of this physiological situation.

The main physiological characteristic of healthy, non-atretic growing follicles is their ability to maintain steroidogenic activity towards follicular dominance. Conversely, the unhealthy atretic follicle shows low E2 production and high P4 concentration in the follicular fluid [15]. Although *in vitro* studies involving cultured GCs have expanded our knowledge on follicular steroidogenesis and development, only GCs in serum-free cultures maintained morphological characteristics of healthy non-atretic growing follicles and maintained their main ability to produce high levels of E2 [16,17]. Furthermore, serum-free cultures represent a robust model in which intraovarian local factors can be precisely and directly evaluated, without the confounding interference of steroids and growth factors.

Although some evidence exists for the role of catecholamines in stimulating ovarian steroidogenesis, it is proposed to study the effects of NE on GC steroidogenesis. Our first objective was to characterize a serum-free bovine GC culture under a chemically defined medium supplemented with polyvinyl alcohol (PVA). Our second and main objective was to identify the effects of various physiological concentrations of NE on the synthesis of P4, E2, and pregnenolone (P3) and so, infer how NE would affect follicular fate in serum-free, gonadotropin-independent conditions.

## Methods

### Culture of bovine granulosa cells

Bovine ovaries from animals from the slaughterhouse were transported on PBS at 37°C, rinsed with 70% ethanol, and rinsed in alpha-MEM (alpha-Minimum Essential Medium, Gibco-BRL, Grand Island NY, USA). Follicles that showed a well vascularized capsule, contained clear follicular fluid, and were between 3 and 5mm in diameter were selected. Thus, most follicles were expected to be non-atretic and to have emerged after a new follicular wave, although prior to the selection of the single dominant follicle. To harvest the GCs, the selected follicles were either aspirated with a 27G syringe or bisected (and gently washed 3 times) in supplemented alpha-MEM. Cells were washed twice using low-speed centrifugation (500g) followed by resuspension in alpha-MEM, and the number and viability of GCs were determined by Trypan blue exclusion. Cell culture procedures were based on a previous work [16], with modifications. Briefly, 500,000 cells/well were seeded into 24-well culture plates in alpha-MEM supplemented with 10mM sodium bicarbonate, 20mM Hepes, antibiotics (1000 U/ml penicillin and 1000 μg/ml streptomycin), 1.4 ng/ml sodium selenite, 5μg/ml transferrin, 0.1 μg/ml insulin, 10 ng/ml human recombinant IGF-1, and 11mM non-essential amino acids. All above reagents were obtained from Gibco BRL (Grand Island NY, USA). In addition, 0.1% PVA (Sigma Chemical Co, St. Louis, MO, USA) was added to adjust oncotic pressure of the medium (usually achieved with the addition of serum) and to avoid steroid contaminants of other serum replacers [18,19]. The E2 precursor, androstenedione (A4), cannot be synthesized by GCs and was also added to the medium (10^-7^ M, Sigma Chemical Co, St. Louis, MO, USA) in physiological conditions. The plates were incubated with 5% CO_2_ at 37°C for 144h (6 days) with change of 70% of the medium every 48h. Media from all experiments were collected and stored at −20°C for posterior analysis of steroid hormones and cholesterol. All experiments were done in accordance to the institutional Research Ethics and Animal Care Committee at School of Medicine of Ribeirão Preto (São Paulo University).

### Experimental design

A total of 3 different sets of culture experiments were designed to determine the effects of norepinephrine in regulating steroid hormone production in bovine GCs. First we characterized the synthesis of steroid hormones and ultrastructural features of the GCs in the chemically defined medium supplemented with PVA. A time-course analysis for E2, P3, and P4 production, as well as cholesterol and A4 concentration and for the E2:P4 ratio was done at 0, 48, 96, and 144h. Based on these results we selected only high E2 cultures to better represent GC from non-atretic growing follicles for the following experiments. Bovine growing follicles are characterized by an increasing production of E2 whereas a pronounced P4 production is only observed before ovulation [15]. Next, dose–response and time-course experiments evaluated NE effects on estrogenically active CGs by the addition of 3 levels of NE concentration (0, 10^-9^, 10^-8^, and 10^-7^M NE [Hipolabor, Belo Horizonte, Brazil]) at different time points: 0, 48, and 96h. Physiological NE concentrations were chosen based on previous studies [14]. Additionally, we evaluated whether the effects of NE occurred via beta-adrenergic receptors in the cultured GCs at 48h by using 10^-8^M propranolol hydrochloride diluted in water (Wyeth-Ayerst Pharmaceuticals, Maidenhead, UK) as a specific beta-adrenergic antagonist. Propranolol was added to the cultures 20 min before NE challenge. Sample collection for all experiments occurred after 1h of NE challenge at the specified time points.

### Hormone and cholesterol assays

Concentrations of E2, A4, P4, and P3 in culture media of GCs were measured with radioimmunoassay using the method described by [20] and modified in our laboratory. The antibodies were generously donated by Dr. Bélanger and Dr. Labrie, CHUL, Quebec, CA. Adequacy of method was evaluated by the parallelism of the slopes from serially diluted medium with the standard curve of each steroid hormone after natural log-logit transformation. The intra-assay and inter-assay coefficients of variation were respectively <5% and <10% for hormone assay. Concentration of total cholesterol in the culture media was evaluated by Trinder-enzymatic assay (COD-ANA Colesterol Liquiform, Labtest Diagnostica SA, Lagoa Santa, Brazil).

### Ultrastructural analysis of granulosa cells

For ultrastructural analysis, GCs were harvested and cultivated as described in the previous section, but at a greater density (10^6^ viable cells/well), in a 6-well plate (Nunc, Roskilde, Denmark) until 96h (when results from hormone production better represented the growing healthy GC). Cells were washed twice in PBS and 0.1M cacodylate buffer (pH 7.4). A total of 20 μl of fixation solution (2% glutaraldehyde, 0.05% calcium chloride, and 2% formaldehyde) was added to the wells for 2h at room temperature. Following fixation, samples were washed twice with 0.1M cacodylate buffer and postfixed in 1% osmium tetroxide, rinsed in double distilled water, dehydrated in increasing concentrations of ethanol, rinsed with acetone, and embedded in resin (Embed 812, EMS, Fort Washington, PA, USA). The resin blocks were cut by ultra-microtomy into 0.5 μm slices. Cellular ultrastructure was observed by a Phillips EM 208 electron microscope (Eindhoven, Netherlands).

### Statistical analysis

All data analyses were performed using SAS software version 6.11 (Statistical Analysis System Institute Inc., Cary, NC, USA). Regression analysis with repeated measurements was performed using MIXED procedures of SAS. The response variables were the production of E2, P4, and P3, the concentration of A4 and cholesterol, and E2:P4 ratio. Final production of hormones (E2, P4, and P3) by GCs was calculated by adding up the concentrations measured at each time. Since only 70% of the medium was changed every time, mathematical calculation were performed to remove the influence of the remaining medium when calculating steroid production over time. In brief, the remaining medium left in the wells was accounted by subtracting 30% of the hormone measured at the previous time in the calculation of the hormone production of the subsequent time point. Based on the characterization experiments, we were able to separate cell cultures into estrogenically active (here called high E2-producing cultures) and estrogenically inactive (low E2-producing cultures) cultures. These two subgroups were included in the statistical model. Thus, the statistical model included experimental culture (estrogenically active or inactive), time, and replicate. Autoregressive correlation coefficient structure for unequally spaced time data was applied when evaluating all time points. Pearson correlation coefficients were used to compare accumulated production levels between hormones that were synthesized by GCs.

The statistical model for the analysis of the effects of different NE levels (0, 10^-9^, 10^-8^, and 10^-7^M) did not used the last time point (144h), based on the results of the GC model characterization experiments. The addition or not of propranolol (at time 48h) was also included in a last regression analysis.

For all experiments, the results represented the least squares means of hormone concentration ± the SEM of at least seven (characterization experiments) or five (NE and propranolol experiments) different experiments performed in triplicate wells. Multiple paired comparisons were adjusted according to Tukey-Kramer. In all analysis, the level of significance was set at p<0.05.

## Results

### Characterization of bovine granulose cells cultured in a chemically defined medium

#### Ultrastructural features of granulosa cells

**Figure 1:**
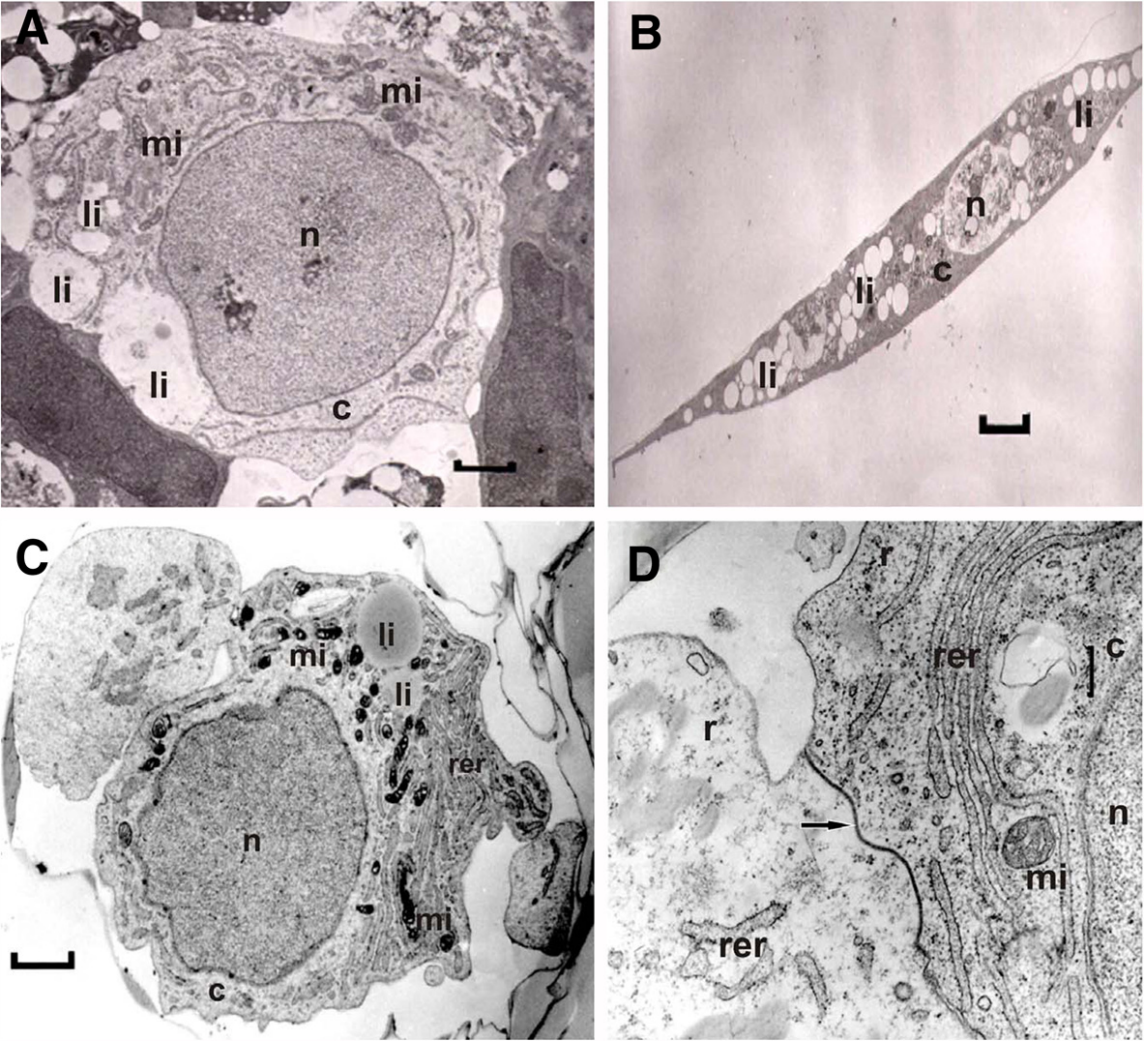
**Electron micrographs of a bovine granulosa cells.** Electron micrographs of a bovine granulosa cells with high nuclear:cytoplasm ratio at 0h of culture (**A**; 0h of culture; magnification: x 6800) and elongated cell with low nuclear:ctoplasm ratio that was randomly found in the culture (**B**; 96h of culture; magnification: x 2720). Note the high concentration of lipid droplets and smaller nucleus in the elongated cell. Bovine granulosa cells presented polyhedral shape, dispersed lipid droplets, and mitochondrias with tubovesicular cristae. (**C**; 48h; magnification bar: x 8500). Neighboring cells within the cluster had rough endoplasmic reticulum, free ribosomes and cellular communication described as tight junctions (**D**; 48h; magnification bar: x 12500). mi = mitochondria; li= lipid droplets; n=nucleus; c= cytoplasm, rer= rough endoplasmic reticulum; r= ribosomes, arrow=tight junctions.

After initial seeding in medium supplemented with 0.1% PVA, bovine GCs aggregated into attached clusters within the first 12h (data not shown). Cluster formation allowed interactions between cells possibly favoring GC polyhedral shape, as well as their high nucleus/cytoplasm ratio and dispersed cytoplasmic lipid droplets, for up to 96h (Figure 1A). Smooth and rough endoplasmic reticulum and free ribosomes were abundant in the cytoplasm of clustered cells, and endoplasmic reticulum was frequently observed in the periphery of the cytoplasm. Mitochondria with tubulovesicular cristae and lipid droplets were randomly distributed through the cytoplasm (Figure 1A and C). Figure 1 (A and C) depicts a high nucleus/cytoplasm ratio cell that was frequently observed in the ultra-thin slices. In contrast, elongated cells (suggestive of fibroblast-like cells, previously described as cells that sustain the cluster of differentiated GC [21]), were not frequently detected and had a low nucleus/cytoplasm ratio and greater amount of lipid droplets (Figure 1B). Cells were attached to each other via desmosomes and tight junctions (Figure 1D).

#### Steroidogenic capacity of granulosa cells

**Figure 2:**
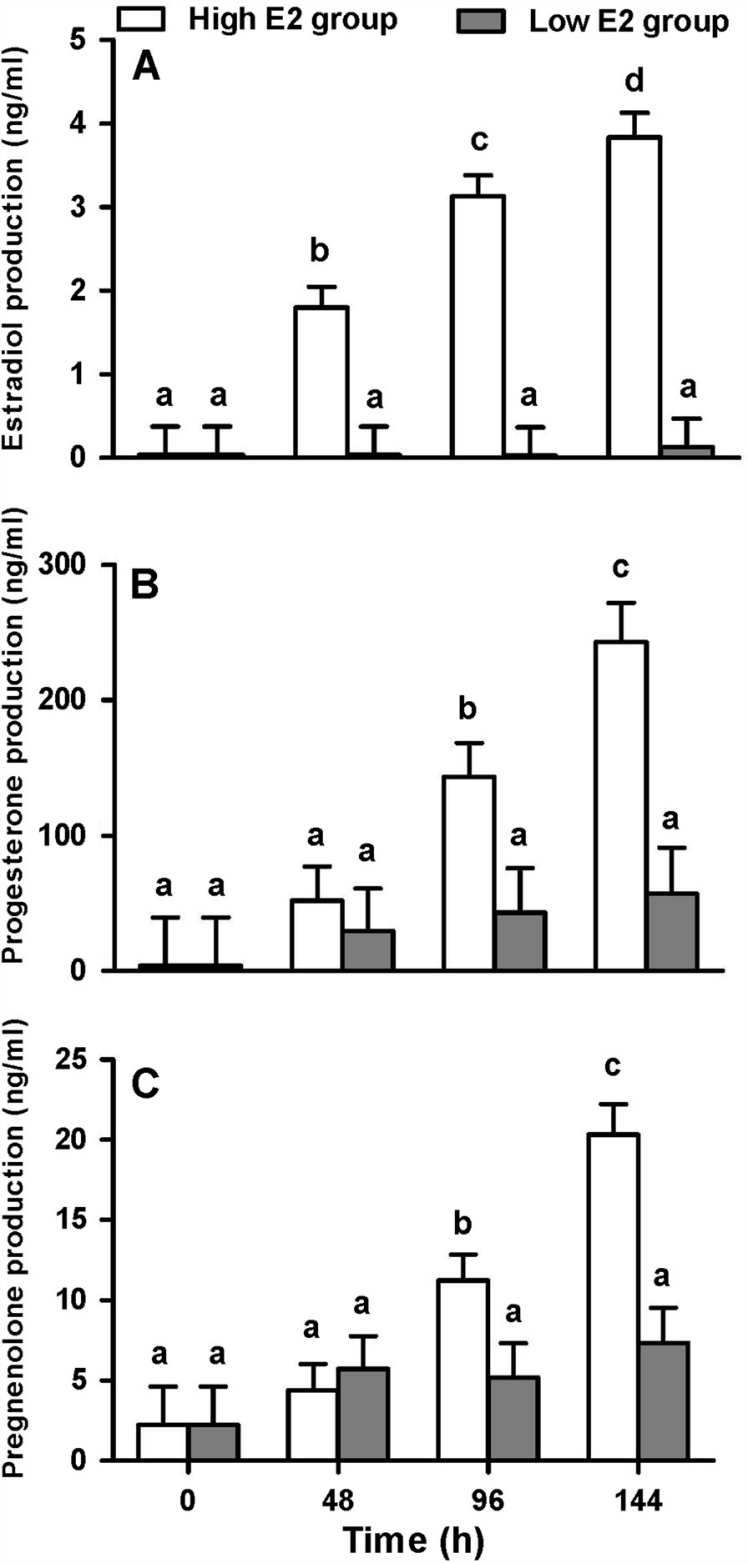
**Estradiol, progesterone, and pregnenolone production by bovine granulosa cells.** Time-effect on production of estradiol (**A**), progesterone (**B**), and pregnenolone (ng/ml) (**C**) by bovine granulosa cells cultivated under chemically defined conditions supplemented with PVA. Values are least square means ± SEM (n=7 independent cultures). ^a, b^ unlike superscripts differ P<0.05.

The steroidogenic capacity towards follicular dominance of GCs in chemically defined medium with PVA was evaluated based on the production of E2, P4, and P3, and on the E2:P4 ratio. Overall, GCs produced increasing amounts of E2 over time. The large variation in the level of E2 production among culture experiments allowed for the classification of two distinct groups of GC cultures: high E2 (E2 ≥ 1ng/ml from 48h and after) and low E2 producers (E2 < 1ng/ml at 48h and after). Such classification permitted the selection of more estrogenically active cultures that better represented the healthy follicle growing towards deviation/dominance. Figure 2A shows E2 production of both groups of GCs cultured under defined conditions.

Estradiol production in high E2-producing cultures reached 1.80 ± 0.25, 3.13 ± 0.25, and 3.84 ± 0.29 ng/ml at 48, 96, and 144h, respectively. The low E2-producing group presented significantly lower E2 concentrations at all evaluated time points (0.04 ± 0.34, 0.029 ± 0.34, 0.13 ± 0.34 ng/ml; P<0.0001). An interaction between groups and time was noted for E2 production (p=0.0038), indicating that the culture of cells with low E2 production did not change over time.

Progesterone production was also greater in high E2-producing cultures (Figure 2B, P=0.014). There was an increase of P4 production over time (P<0.001), and an interaction between time and culture groups was detected (P=0.009). Progesterone production in high E2-producing cultures at 48, 96, and 144h reached 52.1 ± 25.3, 143.2 ± 25.1, and 243.2 ± 28.9 ng/ml, respectively; whereas low E2-producing cultures had lower P4 production (respectively, 29.7 ± 31.5, 43.16 ± 32.9, and 57.2 ± 34.1 ng/ml). However, there was no difference between high E2 cultures and low E2 cultures at 48h (P=0.58). Also, in high E2-producing cultures, P4 production significantly increased only after 96h. Regarding the E2:P4 ratio, only a tendency of culture group effect was detected (P=0.055; data not shown). High E2-producing cultures tended to have greater E2:P4 ratio than low E2-producing cultures at 48h (P=0.07) and 96h (P=0.09), but not at 144h (P=0.42).

Similarly, results of P3 production from GCs under our research conditions indicated an effect of culture groups (P<0.023), time (P<0.0001), and their interaction (P=0.001). Results shown in Figure 2C indicated that P3 production was greater in high E2-producing cultures at 96h (11.24 ± 1.62 vs. 5.19 ± 2.13 ng/ml; p=0.036) and 144h (20.33 ± 1.89 vs. 7.35 ± 2.21 ng/ml; p=0.0003), but not at 48h (4.39 ± 1.64 vs. 5.71 ± 2.04 ng/ml; P>0.05) when compared to low E2-producing cultures.

Interestingly, while there was a significant high positive correlation between E2 and P3 production in high E2-producing cultures (ρ=0.767, P<0.0001), no correlation was detected in low E2-producing cultures (ρ=−0.052, P=0.82). No significant correlation was detected between E2 and P4 production in low E2-producing cultures (ρ=0.37, >0.05) or high E2-producing cultures (ρ=0.482, P>0.05). A positive correlation between P4 and P3 production was detected in high E2-producing cultures (ρ=0.482, P=0.003) and also in low E2 cultures (ρ=0.497, P=0.015). Correlation data can be found in Table 1.

**Table 1:** Correlation and significance between hormone productions

**Group**		**Estradiol**	**Progesterone**	**Pregnenolone**
**High E2 GC**				
	**Estradiol**	--	0.31 (*0.07*)	0.77 (*<.0001*)
	**Progesterone**	0.31 (*0.070*)	--	0.48 (*0.003*)
	**Pregnenolone**	0.77 *(<.0001*)	0.48 (*0.003*)	--
**Low E2 GC**				
	**Estradiol**	--	0.37 (*0.09*)	0.05 (*0.82*)
	**Progesterone**	0.37 (*0.09*)	--	0.49 (*0.015*)
	**Pregnenolone**	0.05 (*0.82*)	0.49 (*0.015*)	--

Correlations and significance between estradiol, progesterone, and pregnenolone productions (ng/ml) by high and low estrogen-producing granulosa cells cultivated under chemically defined conditions.

#### Cholesterol and androstenedione concentration

Cholesterol and A4 were included in the culture medium as precursors of the main follicular hormones. Cholesterol concentrations did not change during 144h of culture and no difference was detected between high and low E2-producing cells (P>0.05). High E2 cultures showed cholesterol concentrations of 64.2 ± 24.5, 42.2 ± 16.3, 64.1 ± 16.2, and 41.7 ± 19.2 μg/ml at times 0h, 48h, 96h, and 144h, respectively; whereas low E2 cultures showed 64.2 ± 24.5, 16.6 ± 24.9, 20.1 ± 30.2, and 25.0 ± 30.5 μg/ml. Similarly, A4 concentrations did not change over time nor between groups (P>0.05). Androstenedione concentrations in high E2 cultures were 46.9 ± 11.7, 27.3 ± 9.22, 33.4 ± 9.2, and 40.5 ± 10.1 ng/ml at times 0h, 48h, 96h, and 144h, respectively. In low E2-producing cells, concentrations of A4 were 46.9 ± 11.7, 53.33 ± 9.1, 57.0 ± 11.9, and 52.0 ± 12.2 ng/ml at the same respective times.

#### Effects of norepinephrine on the steroidogenesis of high E2-producing granulosa cells

Based on our results we established that the high E2 *in vitro* model represents the *in vivo* GCs of a follicle growing towards dominance. The effects of different NE concentrations (0, 10^-9^, 10^-8^, 10^-7^M) on steroid hormone production were then evaluated in the high E2-producing cells for 96h and are shown in Tables 2, 3 and 4. An effect of time was found (P=0.001), but not of NE concentration (P=0.82) or of time*concentration interaction (P=0.72) on E2 production. Progesterone production tended to be affected by NE concentration (P=0.07), but not by time (P=0.61) nor by the interaction between concentration and time (P=0.14). At 48h, the lower concentration of NE (NE 10^-9^ M) had no effect on P4 production (P=0.16), while the higher concentrations of NE (10^-8^ and 10^-7^ M) increased P4 production (P=0.03 and P=0.0007, respectively) when compared to control. A difference in P4 production was also detected between the lower concentration of NE (10^-9^ M) and the higher concentration (10^-7^M, P=0.03). Similarly to what was observed with E2 production, there was an effect of time (P<0.0001), but not of NE concentration (P=0.29) or time by NE concentration (P= 0.91) on P3 production (Table 4).

**Table 2:** Time and dose-dependent effects of norepinephrine on estradiol production

**Time (h)**	**Norepinephrine (M)**			
	**0**	**10**^**-9**^	**10**^**-8**^	**10**^**-7**^
**0**	0.11 ± 0.4^a^	0.11 ± 0.4^a^	0.11 ± 0.4^a^	0.11 ± 0.4^a^
**48**	1.83 ± 0.4^b^	2.61 ± 0.42^b^	2.39 ± 0.4^b^	2.1 ± 0.38^b^
**96**	2.25 ± 0.5^b^	2.25 ± 0.42^b^	2.37 ± 0.4^b^	2.60 ± 0.4^b^

Time and dose-dependent effects of norepinephrine on estradiol (ng/ml) production by highly estrogenic bovine granulosa cells cultivated with 0.1% PVA supplementation.

^a, b^ Means within a column with unlike superscripts differ (*P* < 0.05); values are least square means ± SEM (n=5 independent cultures).

**Table 3:** Time and dose-dependent effects of norepinephrine on progesterone production

**Time (h)**	**Norepinephrine (M)**			
	**0**	**10**^**-9**^	**10**^**-8**^	**10**^**-7**^
**0**	46.9 ± 38.5^a^	46.9 ± 38.5^a^	46.9 ± 38.5^a^	46.9 ± 38.5^a^
**48**	37.0 ± 6.0^ab,A^	125.5 ± 43.0^ab,AB^	171.0 ± 40.5^b,BC^	251.0 ± 38.5^b,C^
**96**	139.9 ± 49.7^b^	175.0 ± 43.0^b^	188.3 ± 43.0^b^	154.8 ± 43.0^ab^

Time and dose-dependent effects of norepinephrine on progesterone (ng/ml) production by highly estrogenic bovine granulosa cells cultivated with 0.1% PVA supplementation.

^a, b^ Means within a column with unlike superscripts differ (*P* < 0.05); ^A, B,C^ Means within a row with unlike superscripts differ (*P* < 0.05); values are least square means ± SEM (n=5 independent cultures).

**Table 4:** Time and dose-dependent effects of norepinephrine on pregnenolone production

**Time (h)**	**Norepinephrine (M)**			
	**0**	**10**^**-9**^	**10**^**-8**^	**10**^**-7**^
**0**	2.1 ± 1.2^a^	2.1 ± 1.2^a^	2.1 ± 1.2^a^	2.1 ± 1.2^a^
**48**	2.5 ± 1.6^a^	4.7 ± 1.4^a^	4.6 ± 1.3^a^	4.4 ± 1.2^a^
**96**	9.0 ± 1.6^b^	12.4 ± 1.4^b^	10.6 ± 1.4^b^	11.9 ± 1.4^b^

Time and dose-dependent effects of norepinephrine on pregnenolone (ng/ml) production by highly estrogenic bovine granulosa cells cultivated with 0.1% PVA supplementation.

^a, b^ Means within a column with unlike superscripts differ (*P* < 0.05); values are least square means ± SEM (n=5 independent cultures).

Cholesterol was already present in the medium and A4 was included in the culture medium. Both steroids are precursors of the main hormones produced by GCs. According to our results, the concentrations of steroid hormone precursor cholesterol and A4 did not change upon NE challenge (P>0.05) nor after 96h of culture (P>0.05) (data not shown).

#### Effects of propranolol on the blockage of NE-mediated steroidogenesis of high E2-producing granulosa cells

**Figure 3:**
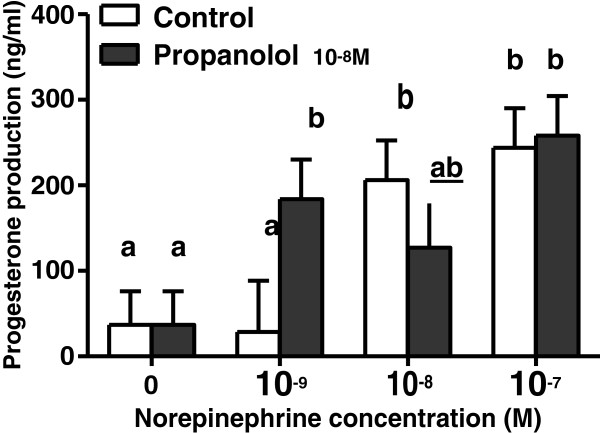
**Propranolol effect on norepinephrine-induced progesterone production.** Effect of propranolol on norepinephrine-induced progesterone production (ng/ml) in highly estrogenic bovine granulosa cells cultivated under chemically defined conditions supplemented with PVA for 48h. Values are least square means ± SEM (n=5 independent cultures). ^a, b^ unlike superscripts differ P<0.05.

The specificity of NE effects on steroidogenesis was investigated by incubating GCs with propranolol (10^-8^M), a non-selective beta-adrenergic antagonist. The greatest accumulation of P4 in media in the presence of NE was observed at 48h of incubation. Consequently, we investigated the effects of propranolol only in regard to P4 production at 48h (Figure 3). At the dose tested, propranolol failed to reverse the effects of NE on P4 secretion (P=0.46), regardless of NE concentration. Unexpectedly, when GCs were exposed to the lower level of NE, propranolol tended to enhance the stimulatory effect of NE (P=0.05). Similarly to our previous results, there was an overall effect of NE levels (P=0.005) on P4 production. There was a ~5.6 fold increase in P4 production (see Figure 3) in the presence of 10^-8^ NE, with no change by propranolol addition. Similarly, regardless of propranolol addition, a 6.6 fold increase in P4 production was detected in the presence of 10^-7^ NE (P= 0.009 and P=0.002, respectively).

## Discussion

The actions of the intraovarian catecholaminergic system have been the focus of a number of studies since the discovery of noradrenergic innervation in the ovarian follicle. The present investigation describes the effects of NE in the steroid hormone profile of a serum-free GC culture system in the context of follicular development and dominance. Our initial aims were to evaluate how effectively the system maintains GC features and follicular steroidogenesis and also, to identify the beta-adrenergic modulation of hormone production. The high E2-producing culture described here represents the ovarian follicle that is growing towards dominance, given that such culture system had greater production of P3, high positive correlation between pro-dominance hormones E2 and P3, and tended to have high E2:P4 ratio. In this physiologically relevant system, it was possible to observe NE stimulation on P4 production, which suggests a pro-luteinization function of NE in the growing follicle.

To our knowledge, the present study is the first to examine the effects of NE on gonadotropin-independent steroidogenesis by primary serum-free GC cultures with high E2 production. The finding that NE is a modulator of follicular steroidogenesis is relevant for a better understanding of local intrafollicular regulatory mechanisms because NE is released by terminal innervations in the outer theca cells and there is evidence of *de novo* synthesis of NE by oocytes [1] and interstitial neuron-like cells [22]. Furthermore, NE binds to adrenergic receptors which are mostly concentrated in other compartments, such as at interstitial and theca cells [23-25]. Thus, NE has the potential to function as an intrafollicular paracrine regulator of GCs. Our finding that NE increased P4 production by bovine GCs in a dose-dependent manner agrees with previous reports that exogenous delivering of NE to cows during the luteal phase [26] and *in vitro* to granulosa-luteal cells [12] enhanced P4 production. However, in both *in vivo* and *in vitro* studies mentioned above, GCs were evaluated during the luteal phase. In cows, during this phase, circulating levels of P4 are already high and follicular E2 production is low. Moreover, these models also presented interfering factors (gonadotropins and serum contaminants, respectively) that prevent a proper evaluation of gonadotropin-independent hormone production. These limitations highlighted the need for a proper and controlled *in vitro* model that allows the investigation of the effects of NE and other putative intraovarian factors in the follicles in a more physiologically relevant state in regards to the modulation of follicular development and dominance.

Steroid hormone production by GCs may vary under different culture conditions. Most serum-based GC culture systems are not adequate for studying the actions of intrafollicular factors because the serum itself already contains an undefined combination of hormones, nutrients, and both growth and attachment factors that frequently leads to GC proliferation [27] and/or luteinization [19]. In fact, E2 production, a pro-dominance marker of GC, declines during culture in serum-coated dishes [18,21]. In the serum-free culture system described here, GCs cultivated under chemically defined medium supplemented with PVA continued to secrete E2 throughout the 144h of culture under gonadotropin-independent conditions. High E2 production by GCs cultured in the presence of bovine serum albumin or other serum replacers has also been described in earlier studies [16,28]. Although attempts to propagate bovine GCs using serum replacers have succeeded in some cases [16,28,29], some of those serum replacers were contaminated with steroids and other interfering factors [19]. Previous experiments from our group suggest that replacement with PVA (and additional defined supplements) allows for greater and earlier E2 production by GCs when compared with bovine serum albumin, a widely used serum replacer [18].

Pregnenolone is an important intermediary metabolite for E2 production and is synthesized at both follicular compartments: granulosa and theca cell [30]. However, only theca cells express the enzymes that convert P3 into androgens, which, in turn, are the direct precursors of E2. Also, the concomitant expression of aromatase (the main E2 synthesizing enzyme) and of cytochrome P450 11A1 (enzyme that converts cholesterol in P3) have been associated to healthy growing follicles greater than 4mm in diameter [31]. The present investigation did not use the expression of steroidogenic enzymes as markers of healthy growing follicles, however a parallel evaluation is proposed here based on the production of E2 and P3, which are the resulting hormones synthesized by these key steroidogenic enzymes associated to growing follicles. In fact, P3 production is a useful marker of active follicular steroidogenesis [30], and an important characteristic of the serum-free culture model described here is the constant increase in P3 and a high significant correlation between E2 and P3 production.

In contrast to findings reported from other bovine GC primary cultures [28,29,32] and even buffalo GC serum-free culture [33], P4 was low during all time points evaluated in our cell cultures and a significant increase was observed only after 96h. A low although increasing P4 production is an important feature of the ideal GC culture system that represents physiological events of an E2 active follicle that is growing towards dominance. Also, the lack of positive correlation between E2 and P4 production reinforces the adequacy of the present culture system.

Our ultrastructural findings show that GCs cultured under the present conditions preserve morphological characteristics similar to those of *in vivo*[34] and *in vitro*[21] E2-producing GCs. Because cellular architecture directly reflects cellular function, culture systems that preserve normal GC morphology are more physiologically relevant [21]. For instance, the maintenance of epithelial characteristics is only possible due to the presence of gap and tight intercellular junctional complexes responsible for intercellular communication [35]. Tight junctions were commonly observed in the GCs in our culture system. Furthermore, elongated mitochondria with tubulovesicular cristae, cytoplasmic lipid droplets, and abundant rough/smooth endoplasmic reticulum were also detected in the GCs and are consistent with features of cells involved in cholesterol and steroid metabolism [34]. Taken together, the steroidogenic profile and the ultrastructural findings of the GC reveal the functional status of the present culture conditions, suggesting direct similarities to the follicle *in vivo* that is growing towards dominance.

Norepinephrine has frequently been studied as an intraovarian modulator of gonadotropins [36,37], prostaglandin [12], and oxytocin [38]. The purpose of the present study, however, was to examine the direct and gonadotropin-independent actions of NE. Our results show a dose-dependent effect of NE in P4 production of high E2-producing bovine GCs. In agreement with this finding, dose-dependent increments in P4 and cAMP were observed in a rat GC model in response to the non-selective beta-adrenergic agonist isoproterenol [39]. Similarly, catecholamines promoted P4 production in *in vitro* porcine GCs [40]. In addition, isoproterenol induced expression of the P4 synthesizing enzyme, 3-beta-hydroxysteroid dehydrogenase, in primary cultures of porcine GC [41]. However, both rat and porcine *in vitro* systems used serum supplementation and consequently, GCs tended to luteinize spontaneously. Thus, those systems present limitations on evaluating the adrenergic effects in the health growing follicle. In contrast, beta-adrenergic agonists minimally stimulated P4 production in hypophysectomized rats [37], perhaps because of *in vivo* interfering factors.

To further substantiate our findings, we evaluated the ability of the non-selective beta-adrenergic antagonist, propranolol, to reverse NE-induced P4 production. It is well established that propranolol acts through beta-1 and beta-2 adrenergic receptors to block catecholaminergic actions. Unexpectedly, propranolol was not able to block P4 production in the lower molar concentrations despite being added at 10-fold molar excess in relation to NE. Although it is possible that propranolol was not added in sufficiently high concentration, the lack of effect seems surprising considering that propranolol reverses isoproterenol-induced increments in P4 production in porcine GCs [40], as well as adrenaline (epinephrine)-dependent P4 stimulation in human GCs [42]. Although an extended dose–response experiment still need to be performed in order to optimize propranolol blockage in the present conditions, a possible explanation to the lack of propranolol effect could be the involvement of alpha-adrenergic receptors mediating NE effects in GCs. Most studies about the effects of intraovarian NE in the presence of non-selective adrenergic agonists and antagonists have suggested that beta-2 adrenergic receptors mediate the effects of NE on P4 production by GCs and luteal cells [9,36,37]. However, NE may also modulate steroidogenesis via alpha-adrenergic receptors [9], or interactions between alpha- and beta-adrenergic receptors. Since most NE-mediated P4 responses have been described in luteal cells or during the luteal phase, we speculate that GCs from developing growing follicles (and not from luteal or luteinized tissues) may have a greater response to NE via alpha-adrenergic receptors. This might be a distinct mechanism from the one previously described in luteal cells. However, this hypothesis still needs to be formally tested in future investigations.

## Conclusions

In conclusion, we employed a strictly defined culture system supplemented with PVA based on a GC culture system described previously [16]. This model is valuable for studying intraovarian factors without the interfering effects of serum or contaminated serum replacers. Our results indicate that catecholamines such as NE may be paracrine/juxtacrine regulators of steroidogenesis and consequently folliculogenesis, by modulating P4 synthesis, and not E2, in the growing follicle. The fate of the follicle could be affected by NE via P4 accumulation that disrupts the follicular balance between E2 and P4, and consequently favors luteinization. The latter observation suggests a role of catecholamines as pro-luteinization factors in the developing follicle, in addition to their already described function in luteal cells. Further understanding of the mechanisms of action of catecholamines in the bovine follicle, as well as the role of the adrenergic system during follicular development, will shed light on the pathogenesis of certain ovarian diseases related to dysfunction of the intraovarian adrenergic systems, such as cystic [14] and anovulatory conditions.

## Competing interests

The authors declare that they have no competing interests.

## Authors’ contributions

CAP carried out the cell cultures, radioimmunoassay and, microscopy, performed the statistical analysis, and drafted the manuscript. CAVC participated in the cell culture and writing. AAV participated in the radioimmunoassay. LHM participated in the design of the study and cell cultures. AAMRS conceived the study, and participated in its design and coordination. All authors read and approved the final manuscript.
